# Service providers’ perspectives on youth sexual and reproductive health services in southern Nigeria: a qualitative interview study

**DOI:** 10.1080/16549716.2026.2758957

**Published:** 2026-11-09

**Authors:** Victoria Kalu Uka, Helen White, Debbie M. Smith

**Affiliations:** a School of Nursing, Public Health and Healthcare Leadership, With Applied Dementia Studies, Faculty of Health and Social Care, University of Bradford, Bradford, UK; b Division of Nursing, Midwifery and Social Work, School of Health Sciences, Faculty of Biology Medicine and Health, University of Manchester, Manchester, UK; c Division of Psychology and Mental Health and Manchester Centre for Health Psychology (MCHP), School of Health Sciences, Faculty of Biology Medicine and Health, University of Manchester, Manchester, UK

**Keywords:** Primary healthcare, health workforce, provider attitudes, sociocultural barriers, gender norms

## Abstract

**Background:**

Despite global commitments to youth-friendly sexual and reproductive health services (SRHS), service delivery gaps persist in sub-Saharan Africa. In Nigeria, health system limitations interact with sociocultural and gender norms, creating barriers to youth SRHS access.

**Objective:**

This study explored service providers’ perspectives on delivering SRHS to youths aged 10–24 years in southern Nigeria, focusing on their experiences, challenges and adaptive strategies.

**Methods:**

A qualitative descriptive study was conducted using semi-structured Zoom interviews with 19 purposively selected service providers from selected public primary healthcare centres (PHCs). Participants came from community health, nursing, midwifery and other clinical backgrounds. Interviews were audio-recorded, transcribed verbatim and analysed using Braun and Clarke’s reflexive thematic analysis.

**Results:**

Four themes were identified: 1) Striving for comprehensive youth SRHS amid resource constraints, reflecting provider commitment despite staffing, infrastructure and supply challenges; 2) Navigating socio-cultural norms in youth SRH provision, showing how taboos, religious beliefs and gender norms shaped delivery; 3) Provider resilience and commitment in a challenging context, reflected in personal sacrifices and creative solutions; and 4) Limited integration of social support services, highlighting missed opportunities for holistic youth support.

**Conclusions:**

Providers demonstrated resilience and resourcefulness, but reliance on individual commitment may mask structural deficiencies and cannot substitute for system-level support. Strengthening youth SRHS requires workforce support, improved infrastructure, standardised youth-friendly protocols, provider training and multisectoral collaboration to improve quality and accessibility. Findings are context-specific to one local government area in southern Nigeria but offer insights for service strengthening in similar low-resource settings.

## Paper Context

- **Main findings**: Service providers described delivering youth sexual and reproductive health services despite persistent workforce shortages, infrastructure gaps, sociocultural pressures and limited referral pathways.
- **Added knowledge**: The study shows how health system constraints, provider commitment, community norms and gendered assumptions shape youth sexual and reproductive health service delivery in a resource-constrained setting.
- **Global health impact for policy and action**: Findings support the need for context-responsive youth health policies that strengthen frontline capacity, non-judgemental care, youth-friendly services and cross-sector collaboration.

## Background

Globally, youths face significant sexual and reproductive health (SRH) challenges, especially in low and middle-income countries (LMICs) where healthcare systems are often under-resourced. An estimated 374 million new sexually transmitted infections (STIs) occur annually, with the highest incidence among those aged 15–24 [1,2]. In sub-Saharan Africa, female youths are more likely than other groups to be affected by human immunodeficiency virus (HIV) and other STIs, have limited access to contraception, and experience more gender-based violence, all of which increase their vulnerability [3–5]

In Nigeria, national data show that nearly two-thirds of unmarried female youths have unmet contraceptive needs, and HIV incidence remains high among youths aged 15–24 [6,7]. Gender-based violence is common, and social norms often prevent open discussion about sexuality [6]. While global guidance encourages comprehensive, youth-friendly care that includes preventative, curative and educational services tailored to the needs of youths [1,2,7,8], putting these into practice remains a challenge in places where resources are stretched. Studies in similar contexts have found that SRHS delivery is often inconsistent, with service quality depending largely on what individual providers are able or willing to do, rather than consistent guidelines [9,10]. These variations are made more complicated by differences in how youth is defined across different programmes and policies.

This study uses the term youth to refer to those aged 10–24 years, following WHO [11] and United Nations [12] definitions. This wide range is increasingly used in research and policy to capture the full span of youth development, as younger and older youths may have different service needs and experiences when accessing SRHS [12–14]. This difference matters because younger and older youths may encounter different service demands, service needs and problems when accessing SRHS. Including the whole age group allows for a more accurate understanding of how SRHS are provided among young people.

To better understand how SRHS are provided to youths, it is necessary to place the study within its local context. The selected local government area is semi-urban, situated in southern Nigeria, and reflects many of the same problems that have been encountered nationwide. The region is culturally varied, with a population estimated to exceed 291,000 in 2022 [15,16]. There is little support for youth-centred care despite a large proportion of youths under 25 years [17]. Due to insufficient midwives and doctors [18,19], Community Health Extension Workers provide most of the care in PHCs, including SRHS for youths, yet their training may not fully prepare them to meet youths’ specific sexual and reproductive health needs [19].

Nigeria’s revised adolescent health policy recognises sexual and reproductive health and rights as a priority area and situates young people’s access to appropriate services within the wider national health and development agenda [20]. The national guidelines for adolescent and youth-friendly health services (AYFHS) further recommend integrating youth-friendly SRH care into primary health care facilities through preventive services, community outreach, curative care and referral pathways [21]. However, implementation remains uneven. A recent study in Calabar Metropolis found that many primary healthcare workers provided youth-friendly SRHS without written guidelines, or any specific training, and did not provide services in a safe space [22]. This policy–practice gap provides important context for the present study. In a recent meta-synthesis, Uka, White and Smith [23] reviewed qualitative studies from sub-Saharan Africa to explore what youths need and desire from SRHS. The findings show that many youths across the region still struggle to access services that are suitable to their age, confidential and delivered in a way that respects their needs. These findings also identified gaps in provider training and communication approaches. This review helped shape the present study, which explores how providers in a semi-urban local government in southern Nigeria understand and respond to these problems in their daily work.

Previous studies have examined youth SRHS access in Calabar and the wider Cross River State, as well as health workers’ knowledge, attitudes and practices (KAP) in relation to youth-friendly SRHS delivery [16,22–25]. However, previous research has not adequately captured how frontline providers negotiate resource constraints, sociocultural expectations and competing clinical responsibilities in the day‑to-day delivery of youth SRHS, or how they adapt when formal support is limited. Providers have an important role in shaping how services are offered and whether youths can access them. This study complements and extends existing youth-focused and KAP-based evidence by exploring the perspectives and experiences of SRHS providers, including the daily challenges they face, the strategies they use and what might support more effective service delivery.

## Methods

### Study design and setting

A qualitative descriptive design was used, guided by the pragmatist and interpretivist paradigms. This approach enabled the exploration of service providers’ subjective experiences, while focusing on the practical implications of service improvement [26,27]. This report was guided by the consolidated criteria for reporting qualitative research (COREQ) 32-item checklist [28] (see supplementary file 1).

This study was conducted in selected public PHCs in one semi-urban local government area in Cross River State, southern Nigeria. The participating facilities serve diverse communities and provide SRHS to youths. Facility names are not reported to preserve anonymity.

### Sample and recruitment

Purposive sampling with criterion and maximum variation strategies was used to select participants [29,30]. Inclusion criteria were healthcare professionals (HCPs) who: 1) were actively involved in youth SRHS delivery; 2) had a minimum 6 months experience in SRHS provision; 3) were currently working in PHCs within a local government area in southern Nigeria; and 4) had willingness to provide informed consent. Participants were recruited through posters distributed by gatekeepers, followed by direct email contact with the researcher after expressions of interest. Two eligible HCPs declined participation due to workload and scheduling conflicts.

The final sample comprised 19 HCPs, with variation in age, sex, professional background and experience in SRHS delivery. Participants were aged 28–58 years. Other participant characteristics are presented in aggregated form in Table 1. Professional roles were grouped, and characteristics were not cross‑tabulated by role, sex or facility to reduce the risk of deductive identification.

**Table 1.** Participant characteristics (*N* = 19).

Characteristics	Category	n (%)
Sex	Female	16 (84.2)
	Male	3 (15.8)
Professional background/role	Community Health Workers (CHEWs and CHOs)	14 (73.7)
	Registered Nurse Midwives	3 (15.8)
	Other Clinical Roles	2 (10.5)
Years of experience in SRH services	0–9 years	6 (31.6)
	10–19 years	9 (47.4)
	≥20 years	4 (21.1)

Consistent with the principles of reflexive thematic analysis, the sample size decision was guided by information power [31]. Given the specific study aim, the use of a focused interview guide, and the richness of data generated from a professionally diverse sample, the sample of 19 participants was considered sufficient. Data saturation was not used, as the study prioritised information relevance and depth over thematic repetition.

### Data collection

Nineteen semi-structured interviews were conducted via Zoom in 2024. The interview guide was informed by findings from a prior meta-ethnographic synthesis on youth needs and preferences regarding SRHS in sub-Saharan Africa [23]. It was pilot tested with two individuals who shared similar characteristics with the study participants to assess clarity and relevance. The guide was structured into six key sections covering various aspects of SRHS provision, including information delivery, service needs, challenges and social support. It consisted of open-ended questions and prompts to explore participants’ experiences in depth. Table 2 summarises the interview topics and provides examples of guiding questions.

**Table 2.** Summary of interview guide topics and sample questions.

S/N	Section	Focus area	Sample questions
1.	Participant background	Role and experience	“Please can you tell us about your role in SRHS delivery to youths”
2.	SRH information	What information is provided and to whom	“What kind of sexual and reproductive health information do you provide to youths?”
3.	Service needs and experience	Types of SRHS and perceived adequacy	Do you feel the current provision meets the needs of youths?”
4.	Service delivery	Challenges and contextual influences	“What are your experiences of delivering SRHS to youths?”
5.	Social needs and support	Social services and unmet needs	“What do you think are the social needs of youths?”
6.	Closing	Additional thoughts or issues not covered	“Is there anything we haven’t covered that you would like to talk about”

All interviews were conducted by the lead researcher (VU), a Nigerian nurse-midwife who conducted the study as part of her PhD and had training in qualitative methods. Her insider perspective, based on familiarity with the local health system and SRHS provision, supported rapport building and contextual sensitivity during data collection. A reflexive diary was maintained to document evolving insights, personal assumptions and contextual observations [32]. No repeat interviews were conducted. Interviews lasted between 55 minutes and 1-hour 59 minutes (mean duration-87 minutes). In-interview member-checking was used, rather than post-interview transcript validation. The researcher regularly verified understanding by summarising participants’ responses and seeking confirmation or clarification when transitioning between interview topics and at the end of the interviews [33].

Before interviews were scheduled, all participants received a participant information sheet (PIS) by email, outlining the study purpose, procedures, confidentiality measures and their rights. This was shared at least 48 hours in advance of the interview to allow time for review. Written informed consent was obtained using the Qualtrics platform, and verbal consent was also confirmed at the start of each interview. All interviews were conducted via Zoom, audio-recorded with consent and transcribed verbatim. This facilitated participation by providers working across different PHCs and enabled interviews to be scheduled around clinical responsibilities without requiring travel. Given the sensitivity of SRH topics, interviews were conducted individually, with no non-participants present, and confidentiality procedures were explained in advance. Rapport was supported by the researcher’s familiarity with the local health system, the use of open-ended questions and in-interview clarification. Although remote interviewing reduced opportunities to observe some non-verbal cues, the richness of data collection was evidenced by detailed accounts of participants’ experiences. Participants received modest compensation (N5,000- Nigerian naira) for their time. To ensure confidentiality, all data were anonymised and securely stored on password-protected systems.

### Data analysis

Interview transcripts were analysed using Braun and Clarke’s six phase reflexive thematic analysis [34,35]. This involved: 1) familiarisation with data through repeated reading; 2) systematic generation of initial codes using NVivo 12; 3) collating codes into potential themes; 4) reviewing themes against the data; 5) defining and naming themes; and 6) producing the report. Codes were developed inductively and grouped into initial themes and sub-themes. These were reviewed against the coded extracts and full transcripts, then refined to ensure each theme captured a distinct pattern in providers’ accounts of youth SRHS delivery. Coding and theme development were led by the first author, with review and guidance from the supervisory team.

### Trustworthiness

Trustworthiness was considered in relation to credibility, dependability, confirmability and transferability [27]. Credibility was supported through in-interview member checking. Participants were given opportunities to confirm or clarify the researcher’s understanding. Credibility was also strengthened by including providers from diverse professional backgrounds and levels of experience.

Dependability was supported by documenting recruitment, data collection, coding and theme development decisions. This provided a clear record of the research process. Confirmability was strengthened through the reflexive diary and data management in NVivo. Participant quotations were also used to show how interpretations were grounded in the data. Supervisory review provided further scrutiny of the developing analysis. Transferability was addressed through description of the study setting, participant characteristics and service context. This provides sufficient contextual information to consider the relevance of the findings to other settings.

## Ethical considerations

Ethical approval was obtained from the University of Manchester (UoM) Research Ethics Committee (Ref: 2023–17475-30669) and a state-level Research Ethics Committee in Nigeria (Ref: CRS/PHCDA/S/71/272). This study was conducted in accordance with the principles of the Declaration of Helsinki. Additional authorisation was granted by the Cross River State Primary Health Care Development Agency (CRSPHCDA), with endorsement from the relevant Local Government Health Authority.

## Results

Through the analytical process, four inter-linked themes were identified, developed and refined. These trace a logical progression from what providers aim to deliver, through the constraints they encountered and the strategies they adopted, to the gaps that remained when individual effort was not sufficient.

Although participants came from different professional backgrounds, the analysis did not set out to identify differences in perspectives by provider cadre. Instead, accounts across provider groups converged around shared challenges, including staffing shortages, limited infrastructure, sociocultural pressures and reliance on individual resourcefulness.

Figure 1 summarises the themes and sub-themes. Verbatim quotations are presented as spoken, with ellipses used to remove non-substantive fillers (example, repetitions or pauses) for clarity. Quotations are labelled using broad professional group codes and anonymous participant numbers: Community Health Worker (CHW), Registered Nurse-Midwife (RNM) and Other Clinical Role (OCR).

**Figure 1.**
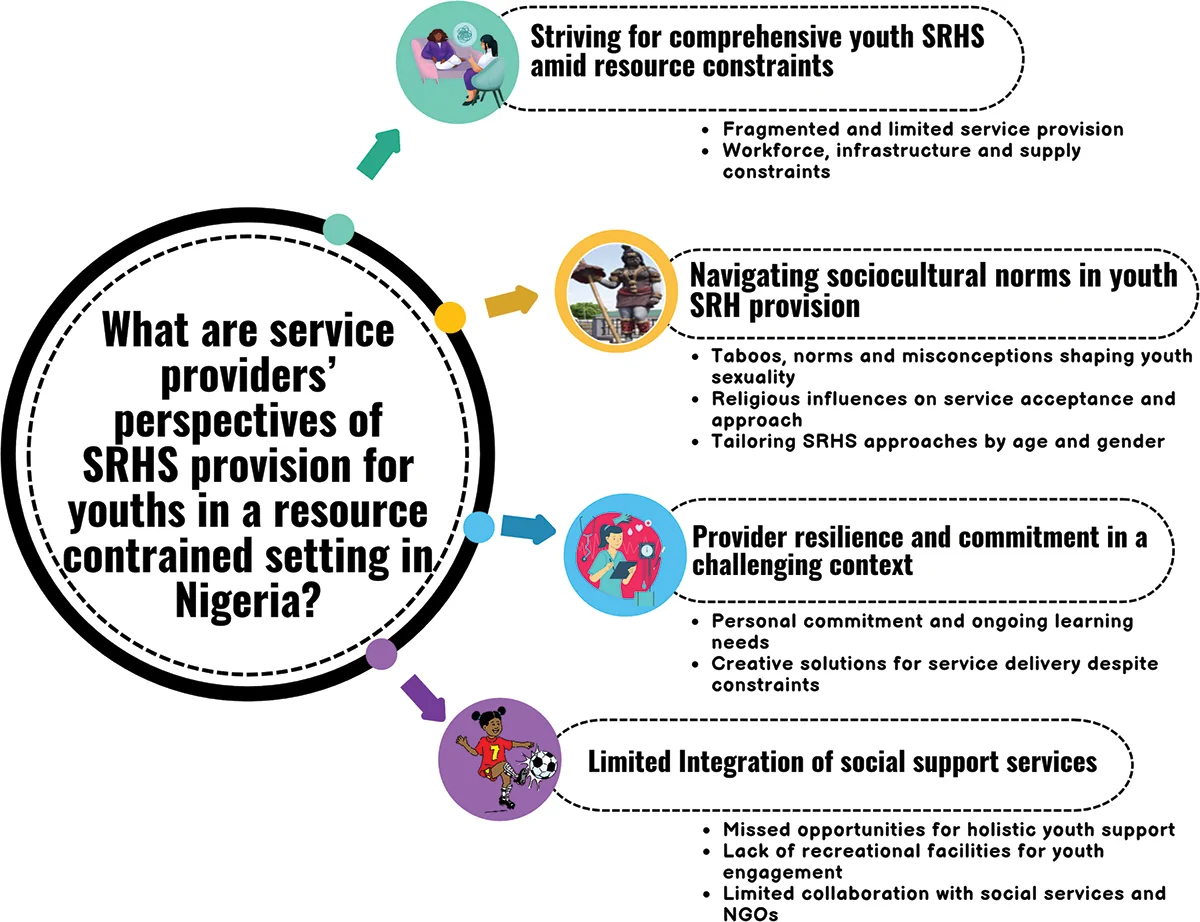
Diagrammatic representation of the themes and subthemes.

### Theme 1: striving for comprehensive youth SRHS amid resource constraints

Service providers were committed to providing a full range of youth SRHS, despite systemic limitations. Their accounts reflected the importance of health education, counselling, contraception and STI management, while also highlighting challenges related to staffing, infrastructure and supply shortages. Two subthemes: *Fragmented and limited services provision, and workforce, Infrastructure and supply constraints* are discussed:

*Fragmented and limited service provision*: Participants described youth SRHS as expected to include health education, preventive care, treatment and referral. In practice, however, service provision was often fragmented and constrained by what was available. A provider described offering *‘health talk to the youths … preventive measures, and … curative [services] and then I do counselling’* (RNM6). Others responded to immediate needs by providing condoms when requested: *‘ … some will come, that I should give condom, I give to them, some will come with their girlfriend … ’* (CHW12). Thus, service provision was shaped by immediate needs, available resources and provider initiative rather than consistently supported service arrangements.

*Workforce, infrastructure and supply constraints*: Providers described staffing shortages alongside infrastructure and essential supplies as constraints on youth SRHS delivery. Understaffing placed considerable pressure on providers, with many describing having to stretch beyond their usual capacity because of vacant posts or inadequate workforce. Some reported working continuously because of staff shortages:*‘ … you will be on night shift and you will still come back and do the morning shift … ’* (CHW7). These workforce pressures occurred within a service environment that was limited by poor infrastructure. Participants described poorly ventilated, overcrowded or inadequately equipped spaces that affected service delivery and provider comfort: *‘ … you go in … , you are sweating like a Christmas goat. I mean, your clothes are wet as a result of sweat … ’* (OCR15).

Providers also reported shortages of basic materials including gloves, STI testing kits and contraceptives. As CHW6 explained, *‘ … we don’t have enough of these commodities like family planning materials’*. Such gaps not only undermined routine services but also reduced the trust youths had in public health facilities. These workforce, infrastructure and supply constraints limited the consistency of youth SRHS and restricted what providers were able to offer. Providers described feeling frustrated and constrained when the staffing, environment and resources needed to deliver services were unavailable.

### Theme 2: navigating sociocultural norms in youth SRH provision

This theme shows how service providers encountered and responded to socio-cultural expectations that shaped how youths accessed SRHS. These expectations influenced not only what services were acceptable but also how providers delivered them. The theme includes three sub-themes*: taboos and misconceptions, religious influences and tailoring services by age and gender.*

*Taboos and misconceptions shaping youth sexuality*: Many providers reported that strong cultural beliefs made it difficult to deliver open, honest SRHS to youths. Within these accounts, sexual activity among unmarried youths was often described as socially inappropriate, leading to reluctance both from some providers and community members to engage openly with the topic. One provider pointed out that *‘ … it is a taboo for a child of … 10 years to 18 years to be actively involved in sex … ’* (RNM13), reflecting a dominant social view that discourages discussing sexual activity.

Some accounts reflected blame towards youths, particularly girls, when discussing sexual violence or pregnancy. As one provider put it, *‘ … some of them, they are moving half naked … So they can be easily raped’* (CHW14). This illustrates tensions between youth-friendly care principles and some providers’ assumptions about youth sexuality, sexual violence and gender norms. It also suggests that providers sometimes internalised community attitudes, potentially shaping how services were offered, discussed or withheld. This may have implications for service quality, confidentiality and youth trust, as young people may avoid services or withhold sensitive information if they anticipate judgement. Providers also perceived that parental restrictions limited youths’ access to SRHS, which may reduce the reach of health education efforts.

*Religious influences on service acceptance and approach*: Religion was another powerful influence, shaping both provider and community attitudes toward SRHS. Some religious leaders were open to health education within their congregations. However, others viewed contraception and open discussions on sex as encouraging immorality. One provider explained that *‘ … when you want to talk about … condom use, it was a bit contrary because … maybe the conclusion was that you’re promoting promiscuity’* (CHW5). Such views meant that even willing providers had to tread carefully when engaging youths.

Several participants made a clear distinction between their personal faith and professional responsibility. One said, *‘Since I’m in the health sector, my religion does not affect it … I used to tell them that they should also do family planning … ’* (CHW14), suggesting that some providers found ways to reconcile personal beliefs with their duty to deliver services. It is worth noting, however, that this view was not universal across the sample. While some providers expressed a clear sense of professional duty that sat alongside their faith, a few others described adjusting their approach in religious settings. For example, one provider explained that during church-based engagement, ‘*most of the method that is advised is on abstinence*’ and that discussing contraception may appear ‘*contradicting*’; therefore, ‘*I focus more on abstinence aspect*’ (CHW1). These conflicting accounts suggest that religion did not shape service delivery in a uniform way. Rather, it influenced how some providers framed SRHS information, especially in religious or community outreach settings.

*Tailoring SRHS approaches by age and gender*: Providers adjusted SRH information based on youths’ age, gender and perceived sexual maturity to improve communication and engagement. Younger youths were typically taught abstinence and basic protective knowledge, while older ones received more practical, risk-reduction advice. One provider explained, *‘The information we give to those who [are] not sexually exposed, will not be the same like those ones who … abuse themselves … ’* (CHW19), suggesting how service providers adapted messages based on their views of youths’ sexual behaviour.

Gender-specific risks also guided how messages were delivered. Some providers portrayed female youths as biologically more vulnerable and at greater risk of sexual violence, with one provider stating, *‘Female own will be differ … their organ is open … ’* (CHW12), reflecting a tendency to frame girls as needing extra caution. In some accounts, male youths were approached with messages rooted in harm-reduction rather than abstinence. One provider stated, *‘ … make sure you are protected … Use your condom … ’* (CHW3), highlighting gendered assumptions that boys are more likely to be sexually active and should therefore be taught to manage, rather than avoid, sexual risk.

In addition to tailoring message content, providers also adapted their communication approach to build rapport and foster trust. Some described using respectful, non-judgemental attitudes as a strategy to help youths feel safe and welcomed. As one put it, *‘ … you welcome them with a smiling face … you don’t carry [family issues] to your place of work … ’* (RNM13), suggesting that a provider behaviour could influence youths’ comfort and willingness to engage with services.

These strategies reflect how both message content and delivery were shaped in response to the diverse realities of youth clients, supporting greater relevance and accessibility of SRHS.

### Theme 3: provider resilience and commitment in a challenging context

While structural constraints created a difficult environment for youth SRHS, this theme explores how individual providers responded with initiative and dedication. Their accounts point to a strong sense of personal responsibility for supporting youths, expressed through unrecognised efforts that went beyond official duties. This included sacrificing personal time, pursuing learning opportunities and developing creative ways to sustain service delivery. Two subthemes are discussed: *personal commitment and ongoing learning needs, and creative solutions for service delivery despite constraints.*

*Personal commitment and ongoing learning needs*: Many providers described taking on additional responsibilities outside of their formal duties. This included covering extra shifts, using personal funds or providing voluntary care outside normal working hours. Some continued to provide care despite financial pressures. For instance, one participant stated: *‘ … I’ve not been paid salary, but I still go in there voluntary … ’* (OCR15). Another explained how the demands of the role strained family life: *‘ … at times when I come my husband would be angry that eh … am I doing two shifts … ’* (CHW2). These accounts reflected the personal demands associated with providers’ commitment to sustaining service delivery.

Providers also recognised the need for ongoing learning to improve youth SRHS delivery. They expressed concern that outdated knowledge and limited opportunities for refresher training could impact their effectiveness. As one provider explained, *‘There is need for retraining … because if the health staff are not equipped … you know, you cannot give what you don’t have … ’* (CHW5). This reflected both a desire for professional growth and a recognition of the evolving needs of youths, especially in areas such as contraception and psychosocial support.

*Creative solutions for service delivery despite constraints*: In the face of equipment shortages and inadequate resources, providers found innovative ways to continue offering care. One described how she maintained hygiene procedures by replacing examination materials using market fabrics: *‘ … I use … one waterproof material that they’re selling in the market now … you’ll buy it like 2, 3 yards. You place it when you finish carrying out the health services, we discard that one … ’* (CHW12). Some leveraged partnerships with non-governmental organisations (NGOs) to sustain service delivery. A participant explained, ‘*There’s an NGO that is supplying us with those consumables … the cotton wool, methylated spirit, even the condom … ’* (RNM6). These efforts allowed providers to keep services running where formal systems fell short.

### Theme 4: limited integration of social support services

This theme examines how SRHS provision in the setting frequently overlooked essential social and psychosocial elements. While the clinical care remained a priority, providers recognised that wider support systems were missing. The three sub-themes here are: *missed opportunities for holistic youth support, lack of recreational facilities and limited collaboration with NGOs and social services.*

*Missed opportunities for holistic youth support*: Providers consistently described youth SRHS as falling short of addressing the broader needs related to psychosocial well-being, life skills and financial empowerment. One provider remarked, *‘Many things are missing oh! Like the different clubs … where they can go and be engaged …* ’ (RNM19), reflecting the absence of complementary programmes that support youth development beyond clinical care. Another linked this gap to adverse outcomes: ‘*There is a lot of stealing … because they are not doing anything … no hope of anything …’* (CHW14).

Some participants also recognised emotional distress in youths, particularly those facing social rejection or hardship. One provider expressed concern that some clients *‘ … behave in a way that … someday they’ll end up committing suicide*’ (CHW16). However, providers reported lacking the training, resources or referral pathways to respond effectively to such psychological needs.

Most participants emphasised the value of vocational training and income-generating opportunities, especially for out-of-school youths. *‘These youths need to be empowered … if government … train them … it will help them … ’* (CHW14), one stated, highlighting how unmet social needs discouraged sustained engagement with SRHS. Although some support was occasionally offered, it was inconsistent. As one provider put it: *‘ … the only social services is … when we have a campaign … ’* (RNM13).

*Lack of recreational facilities for youth engagement*: In addition to limited broader social support, providers also pointed to the scarcity of youth-friendly recreational spaces. These were seen as important for attracting and retaining youth clients and creating safe environments for sensitive discussions. One stated: *‘ … we don’t even have any things for them to do in this centre … ’* (CHW18), while another echoed, *‘we don’t have social activities. Oh, we don’t’* (CHW5).

Although a few centres attempted to share SRH information through media tools like television, *‘ … we have TV … sometimes you put health programmes that they can listen to … ’* (CHW16), these were described as passive and insufficient. More interactive approaches were preferred. As one provider explained, *‘ … we encourage them to come out … and air out their problems … ’* (CHW4), while another added: *‘I make sure that all of them gather once in a while and interact … ’* (RNM13). However, such efforts were limited in scope and lacked consistency. Providers saw the absence of dedicated spaces and structured group activities as a barrier to deeper engagement and peer learning.

*Limited collaboration with social services and NGOs*: Healthcare providers described weak collaboration between health facilities providing SRHS and external bodies like NGOs and government agencies. While some partnerships existed, mainly focused on HIV, they were seen as too narrow. One provider stated, ‘ … *NGO … their own is majorly HIV’* (CHW3), suggesting a limited scope. Another added, *‘Outside condom promotion … that’s the much we can do for now’* (CHW5), further highlighting that support from NGOs was narrow and lacked broader engagement with youth needs.

Some youths benefitted from targeted assistance, particularly those in school, *‘ … they give school bags … just to encourage them …’* (CHW14). Still, these were isolated gestures, not widespread solutions. The majority of providers stressed the need for government coordination to bring stakeholders together. As one put it, *‘ … that collaboration … government should be at the centre and then these other groups would be encouraged … ’* (OCR15). Without structured mechanisms, providers struggled to connect youth with services beyond SRHS.

## Discussion

This study explored how service providers delivered SRHS to youths amid significant constraints. While participants described offering a range of services, including contraception, health education and STI management, these efforts were often sustained through individual commitment and improvised solutions rather than formal structured system support. Providers described making practical adjustments, engaging with communities and tailoring communication to meet the diverse needs of youths in this challenging context.

### Overcoming structural gaps through provider commitment

Despite resource constraints, service providers described delivering a broad range of SRHS, including counselling, contraception, health education and STI treatment. This reflects both the expectations placed on frontline workers and their personal sense of duty, often driven by resilience and improvisation. These findings reiterate research across sub-Saharan Africa showing that SRHS delivery frequently depends on individual commitment rather than formal structures [36,37].

However, shortages of staff, supplies and appropriate infrastructure hindered service consistency. Participants cited a lack of youth-friendly spaces, frequent stockouts and an overstretched workforce, a pattern well documented in the literature [38–40]. The absence of trained SRHS personnel and heavy reliance on under-supported CHEWs reflect a broader neglect of youth health in workforce planning [18,21,41]. Improvisation, such as sourcing materials from local markets or depending on NGO donations, was common, signalling systemic failure. While these strategies reflect resourcefulness, they highlight deep-rooted gaps that require structural solutions through targeted investment in service infrastructure and quality assurance [1,2,38]. These findings suggest that national guidelines for youth-friendly SRHS are essential to ensure quality, equity and consistency. Structured models like the United States’ Title X programme illustrate how clear service standards, supported by dedicated funding, can enhance service quality and accountability [38,42]. Such guidelines alone are not sufficient. Sustainable improvements also depend on strengthened infrastructure, consistent supplies and youth-dedicated spaces. Robust staff support systems require urgent investment to improve service delivery environments and build youth trust. Promoting staff wellbeing has become a growing priority in the United Kingdom’s National Health Service (NHS), recognising its importance for maintaining provider motivation and preventing burnout in under-resourced settings [43].

### Responding to cultural and gender challenges in SRHS delivery

Providers worked with communities where open discussion of youth sexual activity, especially among girls, was seen as taboo. Many expressed difficulties discussing SRH topics due to community disapproval and moral concerns, a finding consistent with studies in conservative settings linking stigma to poor SRHS access [44]. Religion played a dual role: both as a source of resistance and, at times, a pathway for collaboration. Some providers adopted culturally accepted language or worked with religious leaders to deliver messages in ways that aligned with the local norms. While these strategies reflect wider evidence on faith-based engagement in SRH [45,46], they may unintentionally reinforce partial or gender-biased narratives if harmful assumptions about youth sexuality are not directly addressed [47–49].

Gender bias was also evident in how some providers spoke about youths, often blaming girls for sexual violence or pregnancy while overlooking boys’ roles. This echoes broader literature on patriarchal norms shaping SRHS delivery and access [48,49]. These assumptions have important implications for service quality and equity, as youths may experience SRHS as judgemental rather than confidential, supportive and youth-centred. For instance, blaming girls for pregnancy or sexual violence may discourage youths from disclosing sensitive experiences, seeking timely support or returning for care. A few participants did show awareness of gender and age differences, tailoring their engagement accordingly, practices that are reflected in the literature [1,2,7,8,50,51]. However, in the absence of formal policies or training, such efforts remain *ad hoc* and inconsistent.

### Gaps in youth-centred, integrated care models

Providers recognised that youths’ needs go beyond SRH alone. Many described the lack of links to education, psychological support, employment opportunities or safe recreational spaces; resources vital for youth development. This narrow biomedical approach reflects global critiques of SRHS programming in low-income contexts [52–54]. Some providers made efforts to address these needs informally, such as forming peer support groups or giving advice. However, the lack of formal coordination between the health system and sectors like education or social welfare left significant gaps. This supports calls for multisectoral integration in SRHS delivery [55,56]. Integrated models from countries like Zambia and Indonesia show how co-located services and cross-sector collaboration can expand access and improve outcomes [57,58]. Such formal partnerships and referral mechanisms between health, education and social services are therefore needed to ensure integrated support for youths [52,53]. Coordination across sectors may offer more comprehensive care pathways that address the multiple factors affecting youth SRH and wellbeing.

Participants’ accounts also highlighted gaps in psychosocial support and responses to youth vulnerability. Providers recognised the need for psychological support, particularly for youths facing stigma, violence or social exclusion, but described limited tools, structures and support systems to respond effectively. The findings also suggested that some youths may present with signs of emotional distress or social vulnerability during consultations, including potential mental health risks such as suicidal thoughts.

These gaps also point to possible areas for future service development. Existing literature suggests that trauma-informed care may help promote emotional safety, trust and reduce the risk of re-traumatisation [59–61]. Compassion-focused approaches may support non-judgemental responses to shame, stigma and self-blame, while also offering potential benefits for provider wellbeing [62–65]. Digital interventions may provide additional opportunities for confidential SRH information, support and youth engagement [66–73]. However, these were not directly examined in this study and would require context-specific evaluation.

## Strengths and limitations

This study provides valuable insights into the realities of youth SRHS delivery through semi-structured interview exploration of provider perspectives. The use of qualitative methods allowed participants to describe their experiences in detail, highlighting the systemic barriers that might be missed in quantitative assessments. Drawing on experiences from providers across selected public PHCs, the study offers an in-depth understanding of service delivery challenges in this context.

While these findings contribute important knowledge, several factors should be considered when interpreting the results. The study focused on provider perspectives without directly including youth voices, although the study was based on the findings of a systematic review of youth’s experiences. Additionally, findings are based on self-reported data from service providers in a specific semi-urban local government context in Cross River State, southern Nigeria. The cultural and health system characteristics of this setting may differ from other contexts, which should be considered when applying these findings elsewhere.

Despite these considerations, the findings offer important insights for similar resource-constrained settings where providers face comparable challenges in delivering youth SRHS. The detailed accounts of how providers navigate constraints and develop adaptive strategies can inform service strengthening efforts.

## Conclusion

Service providers in this southern Nigerian setting show remarkable dedication in delivering youth SRHS despite severe resource constraints, cultural barriers and limited institutional support. While commitment is commendable, individual efforts cannot substitute for systemic reforms. Priority actions should include strengthening workforce support, improving infrastructure and access to essential supplies. Standardised youth-friendly protocols and provider training are also needed. Training should address cultural competency, non-judgemental communication and awareness of gendered assumptions. Multisectoral referral mechanisms should link health, education and social services. Engagement with religious and community leaders may also support culturally appropriate SRH education and help address stigma surrounding youth sexual health. Future service strengthening could also consider rights-informed, trauma-informed, compassion-focused and digital approaches. As these were not directly examined in this study, they require further context-specific evaluation, including youth participation and appropriate privacy safeguards. The identified barriers and adaptive strategies will be next mapped onto the Capability, Opportunity, Motivation-Behaviour (COM-B) model and Theoretical Domains Framework (TDF) to inform context-specific, theory-informed intervention development. Transforming youth SRHS from a fragmented system dependent on individual provider resilience to one that is comprehensive, well-supported and rights-based will require sustained commitment and coordinated action from governments, development partners and communities. Only through such systemic change can the dedication shown by frontline providers be matched with the structural support needed to ensure equitable access to quality youth SRHS.

## Supplementary Material

COREQ checklist.docx

## Data Availability

The data supporting this study’s findings are not publicly available due to ethical and confidentiality restrictions. Anonymised excerpts are available from the corresponding author upon reasonable request.
