# Supplementary material for: Service providers’ perspectives on youth sexual and reproductive health services in southern Nigeria: a qualitative interview study
Source: Glob Health Action. 2026 Nov 9;19(1):2758957. doi: 10.1080/16549716.2026.2758957 (PMC13757847; doi:10.1080/16549716.2026.2758957)
Supplement: Supplementary file 1 [file ZGHA_A_2758957_SM1580.docx]

**Completed COREQ (Consolidated Criteria for Reporting Qualitative Research) Checklist for: Service providers’ perspectives on youth sexual and reproductive health services in southern Nigeria: A qualitative interview study**

| Item No. | Question | Response | Section Reference |
| --- | --- | --- | --- |
| Domain 1: Research team and reflexivity | | | |
| 1 | Interviewer/facilitator | Primary researcher (VU) conducted all interviews | Methods section |
| 2 | Credentials | PhD researcher | Methods section |
| 3 | Occupation | Nurse and Midwife | - |
| 4 | Gender | Female | - |
| 5 | Experience and training | Qualitative training | Methods |
| 6 | Relationship established | No prior relationship with participants | Methods - |
| 7 | Participant knowledge of the interviewer | Informed via Participant Information Sheet | Methods |
| 8 | Interviewer characteristics | Researcher monitored bias via diary | methods |
| Domain 2: Study design | | | |
| 9 | Methodological orientation | Qualitative descriptive; Reflexive Thematic Analysis | Methods- Design/analysis |
| 10 | Sampling | Purposive sampling with maximum variation | Methods-Sample and recruitment |
| 11 | Method of approach | Email and phone via PHC gatekeepers | Methods - Sample and recruitment |
| 12 | Sample size | 19 participants | Methods - Sample and recruitment |
| 13 | Non-participation | 2 eligible participants declined due to workload | Methods - Sample and recruitment |
| 14 | Setting of data collection | Zoom interviews | Methods - Data collection |
| 15 | Presence of non-participants | None | Methods - Data collection |
| 16 | Description of sample | Community health, Midwifery and other clinical backgrounds; Varied experience | Methods - Sample and recruitment |
| 17 | Interview guide | Semi-structured, pilot-tested | Methods - Data collection |
| 18 | Repeat interviews | None | Methods - Data collection |
| 19 | Audio/visual recording | Zoom audio recording with consent | Methods - Data collection |
| 20 | Field notes | Reflexive diary used throughout interviews | Methods- Data collection |
| 21 | Duration | 55 mins to 119 mins (mean = 87 mins) | Methods - Data collection |
| 22 | Data saturation | Not explicitly used; based on informational power | Methods - Sampling |
| 23 | Transcripts returned | No | Methods - Data collection |
| Domain 3: Analysis and findings | | | |
| 24 | Number of data coders | One (VU), with supervisory guidance | Analysis |
| 25 | Description of the coding tree | Code development and organisation using NVivo 12 | Analysis |
| 26 | Derivation of themes | Inductive from data | Analysis |
| 27 | Software | NVivo 12 | Analysis |
| 28 | Participant checking | No | Analysis |
| 29 | Quotations presented | Verbatim quotes with participant codes | Results |
| 30 | Data and findings consistent | Yes | Results |
| 31 | Clarity of major themes | Clear presentation with sub-themes | Results |
| 32 | Clarity of minor themes | Yes, divergent views included | Results |
